# Effective Admittivity of Biological Tissues as a Coefficient of Elliptic PDE

**DOI:** 10.1155/2013/353849

**Published:** 2013-04-16

**Authors:** Jin Keun Seo, Tushar Kanti Bera, Hyeuknam Kwon, Rosalind Sadleir

**Affiliations:** ^1^Department of Computational Science and Engineering, Advanced Science and Technology Center (ASTC), Yonsei University, 50 Yonsei-Ro, 134 Sinchon-dong, Seodaemun-gu, Seoul 120 749, Republic of Korea; ^2^J. Crayton Pruitt Family Department of Biomedical Engineering, University of Florida, Biomedical Sciences Building JG-5, P.O. Box 116131, Gainesville, FL 32611, USA

## Abstract

The electrical properties of biological tissues can be described by a complex tensor comprising a simple expression of the effective admittivity. The effective admittivities of biological tissues depend on scale, applied frequency, proportions of extra- and intracellular fluids, and membrane structures. The effective admittivity spectra of biological tissue can be used as a means of characterizing tissue structural information relating to the biological cell suspensions, and therefore measuring the frequency-dependent effective conductivity is important for understanding tissue's physiological conditions and structure. Although the concept of effective admittivity has been used widely, it seems that its precise definition has been overlooked. We consider how we can determine the effective admittivity for a cube-shaped object with several different biologically relevant compositions. These precise definitions of effective admittivity may suggest the ways of measuring it from boundary current and voltage data. As in the homogenization theory, the effective admittivity can be computed from pointwise admittivity by solving Maxwell equations. We compute the effective admittivity of simple models as a function of frequency to obtain Maxwell-Wagner interface effects and Debye relaxation starting from mathematical formulations. Finally, layer potentials are used to obtain the Maxwell-Wagner-Fricke expression for a dilute suspension of ellipses and membrane-covered spheres.

## 1. Introduction

The human body can be regarded as a complex electrical conductor comprising many tissues that have distinct electrical properties. Measurements of the electrical properties of biological tissues have shown that effective conductivity (*σ* ^ef^) and permittivity (*ϵ* ^ef^) values of biological tissues in the frequency range from a few Hz to MHz are influenced by physiological and pathological conditions [1–5]. The effective admittivity *γ* ^ef^ = *σ* ^ef^ + *iωϵ* ^ef^ of a biological tissue under the influence of a time-harmonic electric field at an angular frequency *ω* is determined by its ion concentrations in extra- and intracellular fluids, cellular structure and density, molecular compositions, membrane characteristics, and other factors. Cell membranes contribute to capacitance; the intracellular fluid gives rise in an intracellular resistance; the extracellular fluid contributes to effective resistance. As a result, biological tissues show a variable response over the frequency range from a few Hz to MHz. For most biological tissues, *γ* ^ef^ ≈ *σ* ^ef^ at low frequencies below 10 kHz, whereas the *ωϵ* ^ef^ term is not negligible beyond 10 kHz due to the abundant membranous structures in organisms.

The effective admittivity *γ* ^ef^ can be regarded as a function of tissue composition and the applied angular frequency *ω*. Assume that a biological subject under consideration is a mixture of homogeneous tissue at macroscopic length scale and has a constant effective admittivity *γ* ^ef^ in a particular cubic sample voxel. The *γ* ^ef^ can be viewed as the effective tensor according to the well-known concept of homogenization when the admittivity *γ* = *σ* + *iωϵ* is periodic [6]. The effective admittivity *γ* ^ef^ as a function of *ω* and the voxel *V*_oxel_ can be determined by Ohm's law:

(1) $$\begin{array}{c} \int_{V_{\text{oxel}}}J\left(r\right)dr\approx \gamma^{\text{ef}}\left(\omega ,V_{\text{oxel}}\right)\int_{V_{\text{oxel}}}E\left(r\right)dr \end{array}$$

for a time-harmonic electric field **E** and the corresponding current density **J** = *γ* **E** at angular frequency *ω*. Here, *γ* ^ef^(*ω*, *V*_oxel_) is a symmetric 3 × 3 matrix and **r** = (*x*, *y*, *z*) is the position within the voxel. If the quantity (∫_*V*_oxel__ **J**(**r**)*d* **r**)×(∫_*V*_oxel__ **E**(**r**)*d* **r**) ≈ 0 for any pair of electric field **E** and current density **J**, then *γ* ^ef^ is scalar and the subject is isotropic. Otherwise, a subject is said to be anisotropic. Depending on the measurement scale used, anisotropy may or may not be detected. There have been numerous studies and models formulated for admittivity spectra {*γ* ^ef^(*ω*, *V*_oxel_) : 0 ≤ *ω*/2*π* ≤ 100 MHz} of biological tissue as a means of characterizing tissue structural information relating to biological cell suspensions [7, 8]. In 1873, Maxwell [9] derived an expression of *σ* ^ef^  (*ω* = 0) for the special case of a strongly dilute suspension of spherical particles and *ω* = 0. Wagner extended the expression to a general *γ* ^ef^. Poisson [10] in 1826 and Faraday in 1827 dealt with the case of a suspension of infinitely conducting spheres in a background. In 1924, Fricke [7] provided an expression for *γ* ^ef^ by considering the capacity due to a polarization at the interphases or the presence in the interphases of thin poorly conducting membranes. There have been also studies on an effective conductivity of cell suspensions, both analytically and numerically [11–17].

The concept of effective admittivity has been used widely, but it seems that its precise definition has been overlooked. How can we determine the effective admittivity of a given cubic region? In this paper we give precise definitions of effective admittivity to provide a way to measure it from boundary current and voltage data. As in the homogenization theory, the effective admittivity can be computed from pointwise admittivity by solving Maxwell equations. We compute the effective admittivity of simple models as a function of frequency to observe the Maxwell-Wagner interface effect and Debye relaxation using a mathematical point of view. Single layer and double layer potentials are used to produce the Maxwell-Wagner-Fricke expression for a dilute suspension of ellipses and a membrane-covered conductor, respectively. We also note that Maxwell equations make both microscopic and macroscopic senses.

## 2. Effective Admittivity Spectra of Biological Tissues 

The concept of admittivity contains four key definitions: pointwise admittivity, effective admittivity, apparent admittivity, and equivalent admittivity.

- Pointwise admittivity refers to electrical properties at microscopic scale.
- Effective admittivity is defined at macroscopic scale. Homogenization methods have been used to compute effective property of a periodic heterogeneous subject from its pointwise structure [18]. It is used to describe the linear relationship between the ensemble mean current density and the ensemble mean electrical field. Effective admittivity depends only on the electrical properties of the sample.
- Apparent admittivity is defined as the admittivity of electrically homogeneous and isotropic medium that could yield the potential measured on the heterogeneous subject using the same applied current and arrangement of the electrodes.
- Two expressions that have the same effective admittivity are called equivalent admittivity. Pavlin and Miklavčič [19] use a simpler equivalent conductivity of a single cell for the purpose of computation of effective conductivity of a suspension of permeabilized cells.

Let *Ω* be a three-dimensional domain with a pointwise admittivity of *γ*(**r**, *ω*) = *σ*(**r**) + *iωϵ*(**r**), where the conductivity *σ*(**r**) and the permittivity *ϵ*(**r**) values are assumed to depend only on position **r** = (*x*, *y*, *z*), and both are isotropic. Then, the domain *Ω* can be viewed as a union of many voxels *V*_oxel_, and the effective properties mainly depend on the choice of voxels. With a given voxels, we can define the effective admittivity *γ* ^ef^(*ω*) that is a constant on each voxel *V*_oxel_ ⊂ *Ω*. The effective admittivity is a tensor-valued function of the voxel *V*_oxel_ and the angular frequency *ω* such that

(2) γef(Voxel,ω)  =(γxxef(Voxel,ω)γxyef(Voxel,ω)γxzef(Voxel,ω)γxyef(Voxel,ω)γyyef(Voxel,ω)γyzef(Voxel,ω)γxzef(Voxel,ω)γyzef(Voxel,ω)γzzef(Voxel,ω)).

The *γ* ^ef^(*V*_oxel_, *ω*) must be the best approximation of the average of the pointwise admittivity *γ* over the voxel *V*_oxel_ in the sense that

(3) ∫Voxelγ(r,ω)∇u(r,ω)dr≈γef(ω,Voxel)∫Voxel∇u(r,ω)dr     ∀u∈H1(Ω)  satisfying  ∇·(γ(r,ω)∇u)=0  in  Ω.

The physically meaningful solution *u* must have a finite energy [20]:

(4) $$\begin{array}{c} \Phi \left(v\right)=\int_{\Omega }\sigma \left(r\right){\left|\nabla v\left(r\right)\right|}^{2}dr<\infty . \end{array}$$

Hence, the solution of the equation ∇·(*γ*(**r**, *ω*)∇*u*) = 0 should be contained in the set {*v* ∈ *L* ^2^(*Ω*) : Φ(*v*) < *∞*} [20]. Here, *H* ^1^(*Ω*) is the standard Sobolev space equipped with norm $||u||=\sqrt{\int_{\Omega }|\nabla u{|}^{2}+|u^{2}|dr}$.

However, there is no such tensor *γ* ^ef^(*V*_oxel_, *ω*) satisfying (3) exactly. Hence, we may take an appropriate *γ* ^ef^(*V*_oxel_, *ω*) satisfying (3) approximately, and the choice of *γ* ^ef^(*V*_oxel_, *ω*) may differ for a biological sample. To clearly define the effective admittivity, we need to select suitable potentials *u* ∈ *H* ^1^(*Ω*) satisfying ∇·(*γ*(**r**, *ω*)∇*u*(**r**)) = 0 in *Ω*.

### 2.1. Definition of Effective Admittivity for a Cubic Sample

**Figure 1:**
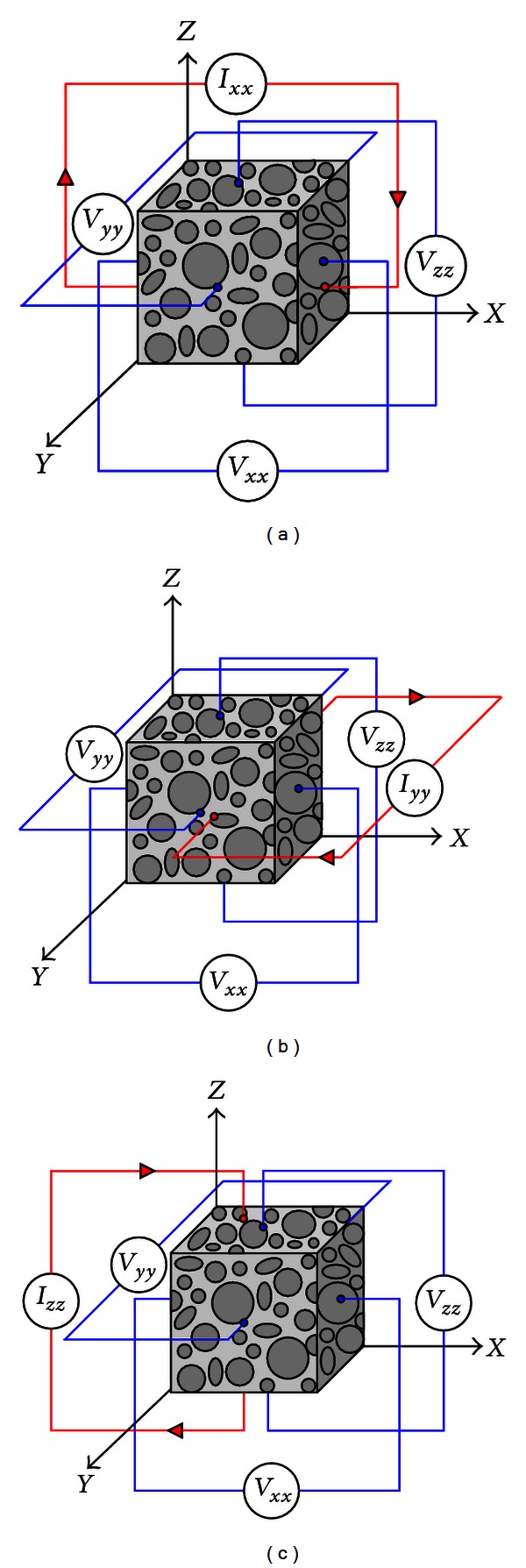
Admittivity measurement of a unit cube of anisotropic material: (a) current injection through *ℰ*_+_^*x*^ and *ℰ*_−_^*x*^ planes, (b) current injection through *ℰ*_+_^*y*^ and *ℰ*_−_^*y*^ planes, and (c) current injection through *ℰ*_+_^*z*^ and *ℰ*_−_^*z*^ planes.

**Figure 2:**
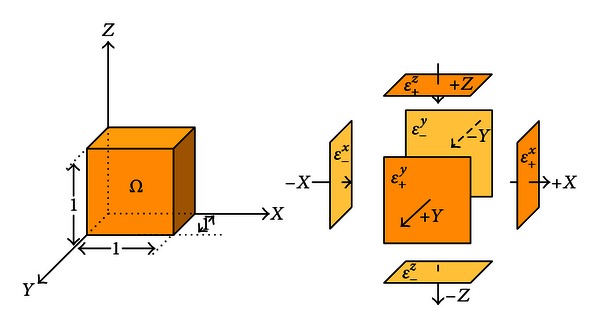
A tissue sample contained in the unit cube.

Let us consider a rectangular-shaped tissue sample (Figure 1) occupied in the unit cube *Ω* = {**r** : 0 < *x*, *y*, *z* < 1 cm} with its three pairs of facing surfaces (Figure 2):

(5) ℰ+x={r∈∂Ω:x=1},  ℰ−x={r∈∂Ω:x=0},ℰ+y={r∈∂Ω:y=1},  ℰ−y={r∈∂Ω:y=0},ℰ+z={r∈∂Ω:z=1},  ℰ−z={r∈∂Ω:z=0}.

Assume that the admittivity distribution of the sample at frequency *ω*/2*π* is given by *γ*(**r**, *ω*) = *σ*(**r**) + *iωϵ*(**r**), where the conductivity *σ*(**r**) and the permittivity *ϵ*(**r**) values are scalar-valued functions depending only on position **r**. Hence, *γ*(**r**, *ω*) is isotropic on a microscopic scale. If we apply a current of *I*(*t*) = *I*_0_cos⁡(*ωt*) through the pair of electrodes attached on *ℰ*_+_^*a*^ and *ℰ*_−_^*a*^, then the resulting time-harmonic potential *u* ^*a*^(**r**, *ω*) satisfies the following equation from a suitable arrangement of Maxwell equations (at frequencies below about 100 kHz):

(6) $$\begin{array}{c} \nabla \cdot \left(\gamma \left(r,\omega \right)\nabla u^{a}\left(r,\omega \right)\right)=0\;\text{for}\;\;r\in \Omega ,\\ n\cdot \left(\gamma \nabla u^{a}\right){\;|\;}_{{ℰ}_{+}^{a}}=I_{0}=-n\cdot \left(\gamma \nabla u^{a}\right){\;|\;}_{{ℰ}_{-}^{a}}\;\left(a\in \left\{x,y,z\right\}\right),\\ n\cdot \nabla u^{a}{\;|\;}_{\partial \Omega \setminus ({ℰ}_{+}^{a}\cup {ℰ}_{-}^{a})}=0, \end{array}$$

where **n** is the unit outward normal vector on ∂*Ω*. For each *a*, *b* ∈ {*x*, *y*, *z*}, we denote the voltage difference *ℰ*_+_^*b*^ by

(7) $$\begin{array}{c} V^{ab}\left(\omega \right)=\int_{{ℰ}_{+}^{b}}u^{a}dS-\int_{{ℰ}_{-}^{b}}u^{a}dS. \end{array}$$

**Lemma 1 (reciprocity)**

For *a*, *b* ∈ {*x*, *y*, *z*}, one has

(8) $$\begin{array}{c} V^{ab}\left(\omega \right)=V^{ba}\left(\omega \right). \end{array}$$

**Proof**

From the boundary conditions of *u* ^*b*^ and divergence theorem, we have

(9) $$\begin{array}{c} V^{ba}\left(\omega \right)=\frac{1}{I_{0}}\int_{{ℰ}_{+}^{b}}n\cdot \left(\gamma \nabla u^{a}\right)u^{b}dS=\frac{1}{I_{0}}\int_{\Omega }\gamma \nabla u^{a}\cdot \nabla u^{b}dr. \end{array}$$

Hence, the symmetry (8) follows from the reciprocity relation

(10) Vab(ω)=1I0∫Ωγ∇ua·∇ubdr=Vba(ω)            ∀a,b∈{x,y,z}.

If ∇*γ*(**r**, *ω*) = 0 (homogeneous), then *V* ^*xx*^(*ω*) = *V* ^*yy*^(*ω*) = *V* ^*zz*^(*ω*) and *V* ^*xy*^(*ω*) = *V* ^*xz*^(*ω*) = *V* ^*yz*^(*ω*) = 0 and *γ* ^ef^(*ω*) must be *γ* ^ef^(*ω*) = *γ*(*ω*) = *I*_0_/*V* ^*xx*^. If the effective admittivity *γ* ^ef^(*ω*) is a diagonal matrix satisfying

(11) (γxxef000γyyef000γzzef)∫Ω∇ua(r,ω)dr=∫Ωγ(r,ω)∇ua(r,ω)dr                        ∀a∈{x,y,z},

then it must be $\gamma_{xx}^{\text{ef}}=\frac{I_{0}}{V^{xx}},\;\;\gamma_{yy}^{\text{ef}}=\frac{I_{0}}{V^{yy}}$, and *γ*_*zz*_^ef^ = *I*_0_/*V* ^*zz*^ due to the following theorem.

**Theorem 2**

If *u* ^*a*^ is the solution of (6), then one has

(12) $$\begin{array}{c} \frac{I_{0}}{V^{xx}\left(\omega \right)}=\frac{\int_{\Omega }\gamma \left(r,\omega \right)\nabla u^{x}\left(r,\omega \right)\cdot \nabla xdr}{\int_{\Omega }\nabla u^{x}\left(r,\omega \right)\cdot \nabla xdr},\\ \frac{I_{0}}{V^{yy}\left(\omega \right)}=\frac{\int_{\Omega }\gamma \nabla u^{y}\cdot \nabla ydr}{\int_{\Omega }\nabla u^{y}\cdot \nabla ydr},\\ \frac{I_{0}}{V^{zz}\left(\omega \right)}=\frac{\int_{\Omega }\gamma \nabla u^{z}\cdot \nabla zdr}{\int_{\Omega }\nabla u^{z}\cdot \nabla zdr}. \end{array}$$

**Proof**

We will only  prove  *γ*_*xx*_^ef^(*ω*). From the definition of *u* ^*x*^, *x*∣_*ℰ*_+_^*x*^_ = 1, and the divergence theorem, we have

(13) I0=∫ℰ+xγ∇ux·ndS=∫ℰ+x(γ∇ux·n)xdS=∫Ωγ(r,ω)∇ux(r,ω)·∇xdr.

Since ∇^2^ *x* = 0 and **n** · ∇*x*∣_*ℰ*_+_^*x*^_ = 1,

(14) Vxx(ω)=∫ℰ+xuxdS−∫ℰ−xuxdS=∫∂Ωux(n·∇x)dS=∫Ω∇ux(r,ω)·∇xdr.

This completes the proof of (12).

Now, we are ready to define the effective admittivity tensor *γ* ^ef^(*ω*).

**Definition 3**

For a given unit cubic *Ω* and each *a*, *b* ∈ {*x*, *y*, *z*}, let *V* ^*ab*^ be the potential difference given in (7). Then the effective admittivity tensor *γ* ^ef^(*ω*) is defined by

(15) [γef(ω)]−1 =(γxxef(ω)γxyef(ω)γxzef(ω)γxyef(ω)γyyef(ω)γyzef(ω)γxzef(ω)γyzef(ω)γzzef(ω))−1∶=1I0(Vxx(ω)Vxy(ω)Vxz(ω)Vxy(ω)Vyy(ω)Vyz(ω)Vxz(ω)Vyz(ω)Vzz(ω)).

The proposed definition may not have coordinate invariance due to its limitation of the tensor expression. For a proper invariance, we need to compute all the tensors (15) by rotating the coordinate system. We may define the effective admittivity tensor as the best fit of the minimization problem described in (3).

Next, we study how the distribution of *γ*(**r**, *ω*) = *σ*(**r**) + *iωϵ*(**r**) is related to the frequency-dependent behavior of {*I* ^*ab*^(*ω*) : 0 ≤ *ω*/2*π* ≤ 10^6^,  *a*, *b* ∈ {*x*, *y*, *z*}}.

### 2.2. One Dimensional Sample

**Figure 3:**
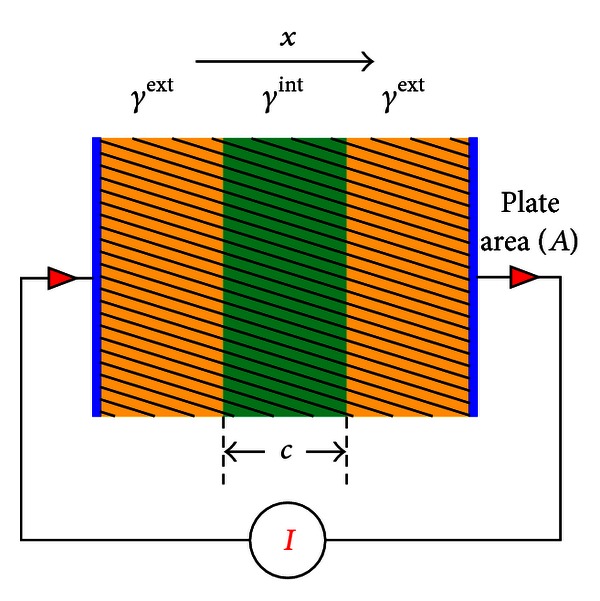
Double layer sandwich type capacitor: a one-dimensional structure.

We begin by considering a special sample (Figure 3) with *γ* depending only on the *x*-variable:

(16) $$\begin{array}{c} \gamma \left(x\right)=\{\begin{array}{cc} \gamma^{\mathrm{int}}=\sigma^{\mathrm{int}}+i\omega \epsilon^{\mathrm{int}} & \text{if}\;\;a<x<a+c\\ \gamma^{\text{ext}}=\sigma^{\text{ext}}+i\omega \epsilon^{\text{ext}} & \text{otherwise}, \end{array} \end{array}$$

where *σ* ^int⁡^,  *ϵ* ^int⁡^,  *σ* ^ext^, and  *ϵ* ^ext^ are constants and 0 < *a* < *a* + *c* < 1. For this sample, the potential *u*(**r**, *ω*) in (6) depends only on *x*-variable, and

(17) $$\begin{array}{c} \frac{d}{dx}\left(\gamma \left(x,\omega \right)\frac{d}{dx}u\left(x,\omega \right)\right)=0\;\text{in}\;\;\left(0,1\right),\\ \frac{d}{dx}u\left(1,\omega \right)=I_{0}=\frac{d}{dx}u\left(0,\omega \right). \end{array}$$

Since *γ*(*x*, *ω*)(*d*/*dx*)*u*(*x*, *ω*) is a constant,

(18) γ(x,ω)ddxu(x,ω)=γ(1,ω)ddxu(1,ω)=I0                for  x∈(0,1).

Writing *V*(*ω*): = *u*(1, *ω*) − *u*(0, *ω*), we have

(19) V(ω)=∫01ddxu(x)dx=∫011γ(x,ω)γ(x,ω)ddxu(x)︸I0dx=I0∫011γ(x,ω)dx.

Hence, it follows from the definition of (15) of *γ* ^ef^ that

(20) $$\begin{array}{c} \gamma_{xx}^{\text{ef}}\left(\omega \right)=\frac{I_{0}}{V\left(\omega \right)}={\left(\int_{0}^{1}\frac{1}{\gamma \left(x,\omega \right)}dx\right)}^{-1}. \end{array}$$

This means that *γ* ^ef^(*ω*) is the harmonic average of the admittivity that can be expressed as

(21) γxxef(ω)=(1−cγext(ω)+cγint⁡(ω))−1=γext(ω)γint⁡(ω)(1−c)γint⁡(ω)+cγext(ω).

From this, we have

(22) γxxef(ω)=σxxef(ω)+iωϵxxef(ω),

(23) σxxef(ω)=σxxef(0)+(σxxef(∞)−σxxef(0))ω2τ21+ω2τ2,ϵxxef(ω)=ϵxxef(∞)+(ϵxxef(0)−ϵxxef(∞))11+ω2τ2,

where

(24) $$\begin{array}{c} \tau =\frac{\left(1-c\right)\epsilon^{\mathrm{int}}+c\epsilon^{\text{ext}}}{\left(1-c\right)\sigma^{\mathrm{int}}+c\sigma^{\text{ext}}},\\ \sigma_{xx}^{\text{ef}}\left(0\right)=\frac{\sigma^{\text{ext}}\sigma^{\mathrm{int}}}{\left(1-c\right)\sigma^{\mathrm{int}}+c\sigma^{\text{ext}}},\\ \epsilon_{xx}^{\text{ef}}\left(\infty \right)=\frac{\epsilon^{\text{ext}}\epsilon^{\mathrm{int}}}{\left(1-c\right)\epsilon^{\mathrm{int}}+c\epsilon^{\text{ext}}},\\ \sigma_{xx}^{\text{ef}}\left(\infty \right)=\frac{\sigma^{\mathrm{int}}\epsilon^{\text{ext}}+\sigma^{\text{ext}}\epsilon^{\mathrm{int}}}{\left(1-c\right)\epsilon^{\mathrm{int}}+c\epsilon^{\text{ext}}}-\frac{\epsilon^{\text{ext}}\epsilon^{\mathrm{int}}}{\left(1-c\right)\sigma^{\mathrm{int}}+c\sigma^{\text{ext}}},\\ \epsilon_{xx}^{\text{ef}}\left(0\right)=\frac{\sigma^{\mathrm{int}}\epsilon^{\text{ext}}+\sigma^{\text{ext}}\epsilon^{\mathrm{int}}}{\left(1-c\right)\sigma^{\mathrm{int}}+c\sigma^{\text{ext}}}-\frac{\sigma^{\text{ext}}\sigma^{\mathrm{int}}}{\left(1-c\right)\epsilon^{\mathrm{int}}+c\epsilon^{\text{ext}}}. \end{array}$$

Writing Δ*σ*_*xx*_^ef^ = *σ*_*xx*_^ef^(*∞*) − *σ*_*xx*_^ef^(0) and Δ*ϵ*_*xx*_^ef^ = *ϵ*_*xx*_^ef^(*∞*) − *ϵ*_*xx*_^ef^(0), we have

(25) $$\begin{array}{c} \Delta \epsilon_{xx}^{\text{ef}}=\tau \Delta \sigma_{xx}^{\text{ef}}. \end{array}$$

Here, *τ* is referred to as a relaxation time, since its value controls polarization time [8, 21]. It is remarkable to observe that the relaxation time *τ* = ((1 − *c*)*ϵ* ^int⁡^ + *cϵ* ^ext^)/((1 − *c*)*σ* ^int⁡^ + *cσ* ^ext^) may be obtained by solving the elliptic PDE (17).

Using (23), the average current density **J** = −∫_0_^1^ *γ*∇*u* generated inside the dielectric due to the average electric field **E** = −∫_0_^1^∇*u* is given by

(26) $$\begin{array}{c} J=\left(\sigma_{xx}^{\text{ef}}\left(\omega \right)+i\omega \epsilon_{0}\epsilon_{xx}^{\text{ef}}\left(\omega \right)\right)E, \end{array}$$

which can be expressed as

(27) $$\begin{array}{c} J=\sigma_{s}E+i\omega \epsilon_{0}\left(\epsilon_{xx}^{\prime }\left(\omega \right)-i\epsilon_{xx}^{\prime \prime }\left(\omega \right)\right)E, \end{array}$$

where *σ*_*s*_ = *σ*_*xx*_^ef^(0) and

(28) $$\begin{array}{c} \epsilon_{xx}^{\prime }\left(\omega \right)=\epsilon_{xx}^{\text{ef}}\left(\infty \right)+\left(\epsilon_{xx}^{\text{ef}}\left(0\right)-\epsilon_{xx}^{\text{ef}}\left(\infty \right)\right)\frac{1}{1+\omega^{2}\tau^{2}},\\ \epsilon_{xx}^{\prime \prime }\left(\omega \right)=\frac{1}{\omega \epsilon_{0}}\left(\left(\sigma_{xx}^{\text{ef}}\left(\infty \right)-\sigma_{xx}^{\text{ef}}\left(0\right)\right)\frac{\omega^{2}\tau^{2}}{1+\omega^{2}\tau^{2}}\right). \end{array}$$

Here, *ϵ*_*xx*_′(*ω*) and *ϵ*_*xx*_′′(*ω*) are referred to as the dielectric constant and loss factor of the dielectric material, respectively. The average current density can also be written as

(29) $$\begin{array}{c} J=\underset{\sigma^{\text{ef}}(\omega )}{\underset{︸}{\left(\sigma_{s}+\omega \epsilon_{0}\epsilon_{xx}^{\prime \prime }(\omega )\right)}}E+i\omega \underset{\epsilon^{\text{ef}}(\omega )}{\underset{︸}{\epsilon_{0}\epsilon_{xx}^{\prime }(\omega )}}E. \end{array}$$

In biological materials *σ*_*s*_ is produced by the ionic conduction and *ωϵ*_0_ *ϵ*_*xx*_′′(*ω*) is produced by dielectric relaxation.

The dielectric response of biological tissues is always frequency dependent, and the electric charge movement inside the material in response to an externally applied electric field is controlled by the dielectric properties of the material. The free charge movement inside a material affected by an external field is controlled by its conductivity (*σ*). Biological tissues display extremely high dielectric constants at low frequencies, and as the excitation frequency is increased, the dielectric constants of the tissues fall off in more or less distinct steps [8]. Interfaces play a significant role in the frequency dependence of complex materials, particularly at audio and subaudio frequencies [8]. The frequency response of biological tissue admittivity is highly influenced by the dielectric polarization, dielectric relaxation, and dielectric dispersion.

**Figure 4:**
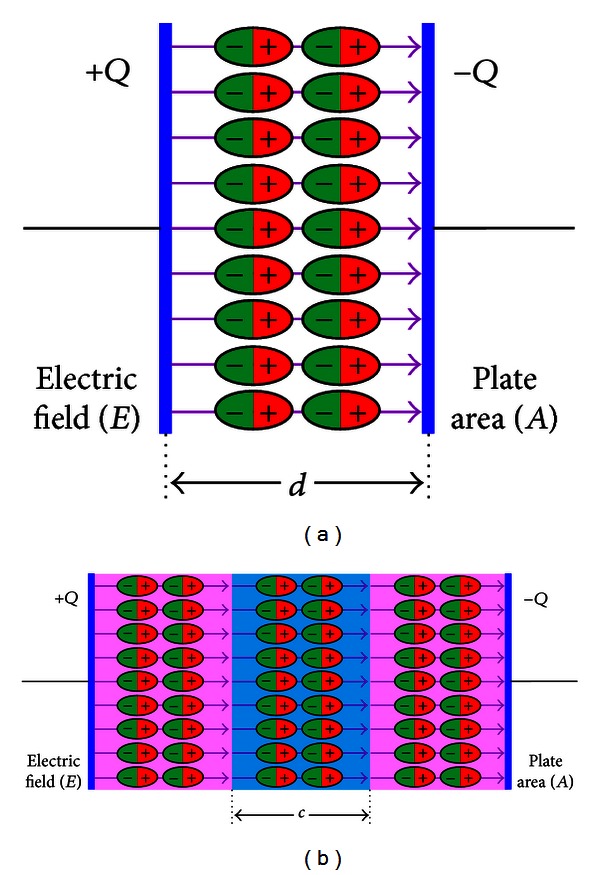
Dielectric polarization inside capacitors under an electric field *E*: (a) single dielectric capacitor and (b) double dielectric capacitor.

**Figure 5:**
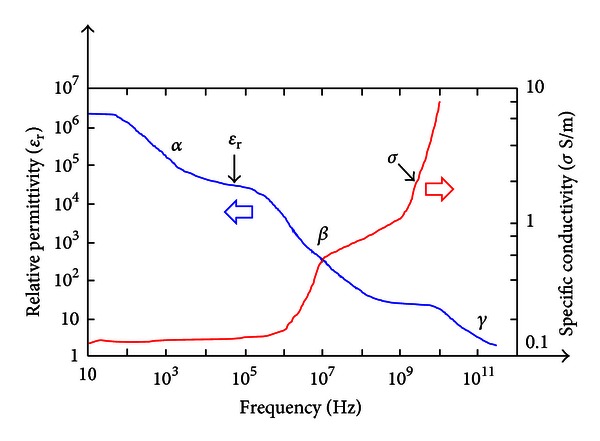
The variations of the complex permittivity of the biological tissues with frequency.

Electric polarization (Figure 4) may be defined as the electric-field-induced disturbance (shift from average equilibrium positions) of the charge distribution in a region [8]. Dielectric dispersion in biological tissues can be assumed to depend upon the permittivity (Figure 5) of tissue material with applied electric field frequency [8]. In other words, a significant change in dielectric properties over a frequency range, by convention, is called a dielectric dispersion [21].

As there is always a lag between the changes in an applied electric field and changes in polarization, the permittivity of the biological tissues is a complex-valued function of the frequency of the applied electric field. The term dielectric relaxation [22] in a biological tissue connotes the delay or lag in its response to create the dielectric polarization following the application of electric field across the tissue sample. In other words, the dielectric relaxation of a tissue can be defined as the lag (momentary delay) in the dielectric constant which is usually caused by the delay in molecular polarization with respect to a change in applied electric field. According to the previous simple computations of (23) in the 1D model (17), the central frequency of the dispersion is   *f*_*c*_ = 1/2*πτ* = (1/2*π*)((1 − *c*)*σ* ^int⁡^ + *cσ* ^ext^)/((1 − *c*)*ϵ* ^int⁡^ + *cϵ* ^ext^).

Schwan [23, 24] studied the properties of biological tissue and cell suspensions over a large frequency range and observed that the dielectric properties of biological tissues are characterized by three major dispersions, *α*-dispersion [23, 24], *β*-dispersion [23, 24], and *γ*-dispersion [23, 24] occurring at low frequency, radio frequency, and microwave frequency, respectively. We consider each of these dispersions below.

- *α-dispersion *(10 Hz ≤ *ω*/2*π* ≤ 10 kHz): The *α*-dispersion is associated with tissue interfaces such as membranes [23]. Below about 10 kHz, the dielectric studies of biological or any other electrolyte systems become very complex and difficult to characterize. Foster and Schwan, 1989 [25], reported that *α*-dispersion is believed to be associated with a counterion layer (electrical double layer) polarization in tissues.
- *β-dispersion *(10 kHz ≤ *ω*/2*π* ≤ 10 MHz): In biological tissues, the *β*-dispersion is caused by the polarization of cellular membranes and polarization of protein and other organic macromolecules [23]. The *β*-dispersion arises, principally, from interfacial polarization (Maxwell-Wagner effect) [26] of cell membranes [21]. In the frequency range 10 kHz ≤ *ω*/2*π* ≤ 10 MHz, the dielectric behavior of the tissues is dominated by the heterogeneous composition and ionic activities inside the biological tissue. These effects are principally responsible for the *β*-dispersion. The radio frequency dispersion or *β*-dispersion has been recognized as a Maxwell-Wagner relaxation [26] caused by cell membranes [27]. A large magnitude, low frequency *β*-dispersion was observed by Schwan [28] in a muscle tissue. This effect is related, in part at least, to the tubular shape of muscle fibers [29]. The theoretical aspects of the low frequency dispersion of colloid particles in electrolyte solution have been studied by Schwarz in 1962 [30].
- *γ-dispersion *(*ω*/2*π* ≥ 10 GHz). The *γ*-dispersion in biological tissues is caused by the reorientation of water molecules [18]. This dispersion has been well studied and has found many applications [31–33]. Rajewsky and Schwan [34] noted the *γ*-dispersion at microwave frequencies which is understood to be caused by abundant tissue water. Schwan conducted the extensive studies on the electrical properties of biological cell suspensions in 1993 [35] over a broad frequency range extending from less than 1 Hz to many GHz and summarized the mechanisms which contribute to the total frequency response. He studied the mechanisms responsible for electrical properties of tissues and cell suspensions, and he observed that the frequency changes of these properties obey causality, that is, the Kramers-Kronig relationships [35] which relate changes of dielectric constants to conductivity changes. A number of mechanisms which reflect the various compartments of the biological materials were identified such as membranes and their properties, biological macromolecules, and fluid compartments inside and outside membranes.

Membrane relaxation is anticipated from the Hodgkin-Huxley membrane model [36] and adds to the *γ*-effects [8], and hence a number of *β*-effects of small magnitude occur at the tail of the *β*-dispersion caused by proteins, proteinbound water (called *δ*-dispersion), and cell organelles such as mitochondria [37]. A second Maxwell-Wagner dispersion [26] which occurs at frequencies well above those of the main *β*-dispersion [8] is a characteristic of suspended particles surrounded by a shell and usually of small magnitude [14].

**Figure 6:**
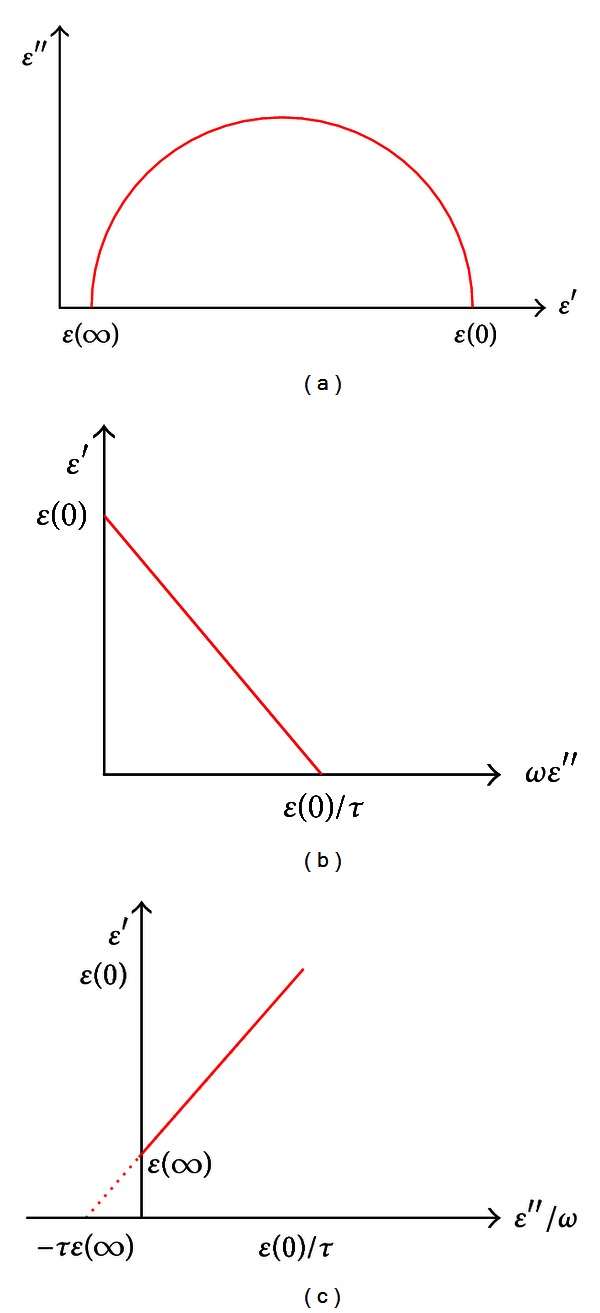
Dielectric dispersion curves: (a) Cole-Cole plot, (b) *ϵ*′ versus *ωϵ*′′ line, and (c) *ϵ*′ versus *ϵ*′′/*ω* line.

Figure 6(a) shows Cole-Cole plot explaining (*ϵ*′ + (*ϵ*_*xx*_^ef^(*∞*) + *ϵ*_*xx*_^ef^(0))/2)^2^ + (*ϵ*′′)^2^ = ((*ϵ*_*xx*_^ef^(0) − *ϵ*_*xx*_^ef^(*∞*))/2)^2^.  Figure 6(b) shows *ϵ*′ versus *ωϵ*′′ line describing *ϵ*′ = *ϵ*_*xx*_^ef^(0) − *τ*(*ωϵ*′′).  Figure 6(c) shows *ϵ*′ versus *ϵ*′′/*ω* line.

**Remark 4**

In the case when *c* is sufficiently small (dilute suspension) so that |1 − *γ* ^ext^/*γ* ^int⁡^|*c* ≪ 1, (21) can be expressed as

(30) $$\begin{array}{c} \gamma_{xx}^{\text{ef}}\left(\omega \right)=\gamma^{\text{ext}}\left(\omega \right)\left(1+\left(1-\frac{\gamma^{\text{ext}}\left(\omega \right)}{\gamma^{\mathrm{int}}\left(\omega \right)}\right)c+O\left(c^{2}\right)\right) \end{array}$$

because *γ* ^ef^(*ω*) = *γ* ^ext^(*ω*)/(1 − (1 − *γ* ^ext^(*ω*)/*γ* ^int⁡^(*ω*))*c*). Neglecting *O*(*c* ^2^) in (30), we get

(31) γef(ω)≈(γint⁡(ω)−(γext(ω)−γint⁡(ω))c)γext(ω)γint⁡(ω)=(σint⁡+c(σint⁡−σext)+iω[ϵint⁡+c(ϵint⁡−ϵext)])×σext+iωϵextσint⁡+iωϵint⁡.

In three dimensional heterogeneous medium, this type of dilute suspension model with neglecting *O*(*c* ^2^) had been used in computation of the effective admittivity *γ* ^ef^.

Next, we will investigate the effective admittivity for dilute suspensions of membrane of materials. We will express potential of models comprising suspension of arbitrary-shaped membrane, using double layer potential technique.

### 2.3. Dilute Single Suspension of Ellipses in a Cube

Maxwell [9] and Wagner [38] analyzed expressions for the effective admittivity *γ* ^ef^ of a strongly diluted suspension of spheres [7].

**Figure 7:**
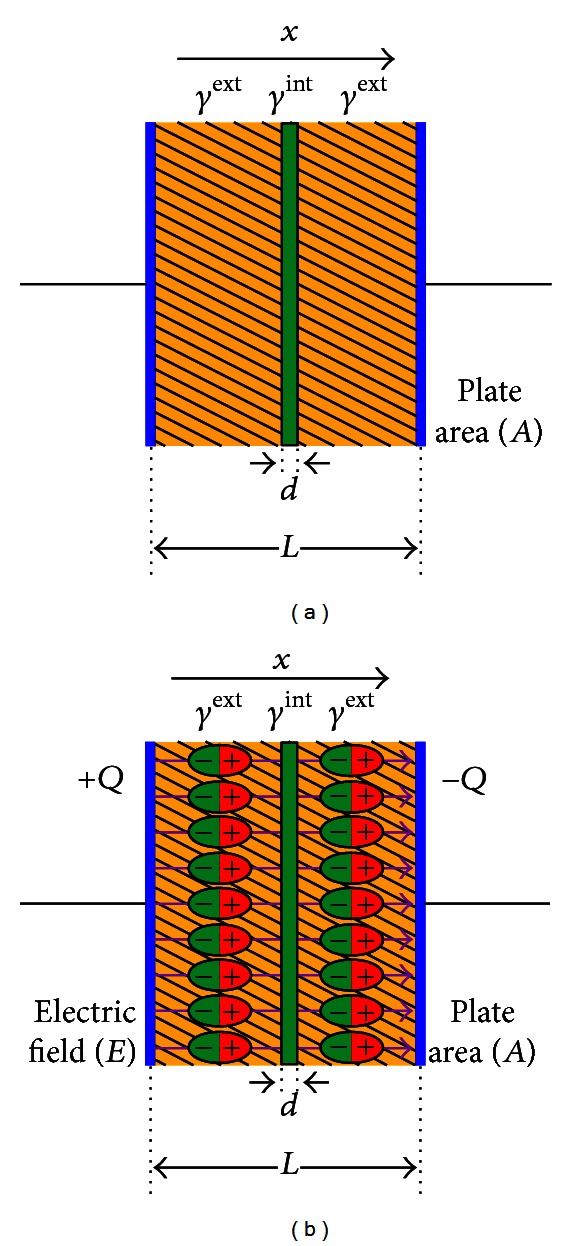
Dielectric phenomena inside a capacitor or a dilute suspension of a thin membrane, (a) capacitor with a thin membrane of thickness *d*, (b) dielectric polarization under an electric field *E*.

Let *Ω* = {**r** : −1 < *x*, *y*, *z* < 1}  be a cube, and let *D* = {**r** ∈ *Ω* : *x* ^2^/*a*_1_^2^ + *y* ^2^/*a*_2_^2^ + *y* ^2^/*a*_3_^2^ < 1} be an ellipsoid with 0 < *a*_1_ ≤ *a*_2_ ≤ *a*_3_ ≪ 1. As in the previous section, let the admittivity distribution *γ* (Figure 7) be given by

(32) $$\begin{array}{c} \gamma \left(r\right)=\{\begin{array}{cc} \gamma^{\mathrm{int}}=\sigma^{\mathrm{int}}+i\omega \epsilon^{\mathrm{int}} & \text{for}\;\;r\in D\\ \gamma^{\text{ext}}=\sigma^{\text{ext}}+i\omega \epsilon^{\text{ext}} & \text{for}\;\;r\in \Omega \setminus D. \end{array} \end{array}$$

If *u* ∈ *H* ^1^(*Ω*) is a potential satisfying ∇·(*γ*(**r**, *ω*)∇*u*(**r**, *ω*)) = 0 in *Ω*, then it can be expressed as a sum of harmonic function *H*(**r**, *ω*) in *Ω* and a single layer potential:

(33) u(r,ω)=H(r,ω)+∫∂D14π|r−r′|ϕ(r′,ω)dsr′                   for  r∈Ω,

where *ϕ* is determined by

(34) (γext+γint⁡2(γext−γint⁡)I−𝒦D∗)ϕ(r,ω)=n(r)·∇H(r,ω)                  for  r∈∂D,𝒦D∗ϕ(r,ω)=∫∂D〈r−r′,n(r)〉4π|r−r′|3ϕ(r′,ω)dsr′                  for  r∈∂D.

Assuming that the volume fraction *c* = |*D*|/|*Ω*| is small, Fricke obtained the following approximation:

(35) $$\begin{array}{c} \gamma_{xx}^{\text{ef}}\approx \gamma^{\text{ext}}I+c\left(\gamma^{\mathrm{int}}-\gamma^{\text{ext}}\right)\left(\begin{array}{ccc} \beta_{1} & 0 & 0\\ 0 & \beta_{2} & 0\\ 0 & 0 & \beta_{3} \end{array}\right), \end{array}$$

where

(36) βj=1+ξjξj+γint⁡/γext,ξj=2−a1a2a3Lja1a2a3Lj,Lj=∫0∞dλ(aj2+λ)(a12+λ)(a22+λ)(a32+λ)                 (j=1,2,3).

When *α*_*x*_ = *α*_*y*_ = *α*_*z*_ (*D* is sphere) and the volume fraction *c* = |*D*|/|*Ω*| is small, Maxwell-Wagner [26] formula for *γ* ^ef^(*ω*) is given by

(37) γef(ω)=(σext+iωϵext)×(1+3c(σint⁡−σext)+iω(ϵint⁡−ϵext)(σint⁡+2σext)+iω(ϵint⁡+2ϵext))+O(c2).

With the aid of *τ* = (*ϵ* ^int⁡^ + 2*ϵ* ^ext^)/(*σ* ^int⁡^ + 2*σ* ^ext^), we can derive the Debye dispersion function for *γ* ^ef^(*ω*) as follows:

(38) γef(ω)=σef(∞)+(σef(0)−σef(∞))ω2τ21+ω2τ2︸σef(ω)+iω[ϵef(∞)+ϵef(0)−ϵef(∞)1+ω2τ2]︸ϵef(ω).

Next, we will investigate the effective admittivity for dilute suspensions of membranes.

### 2.4. Dilute Single Suspension of Membrane

**Figure 8:**
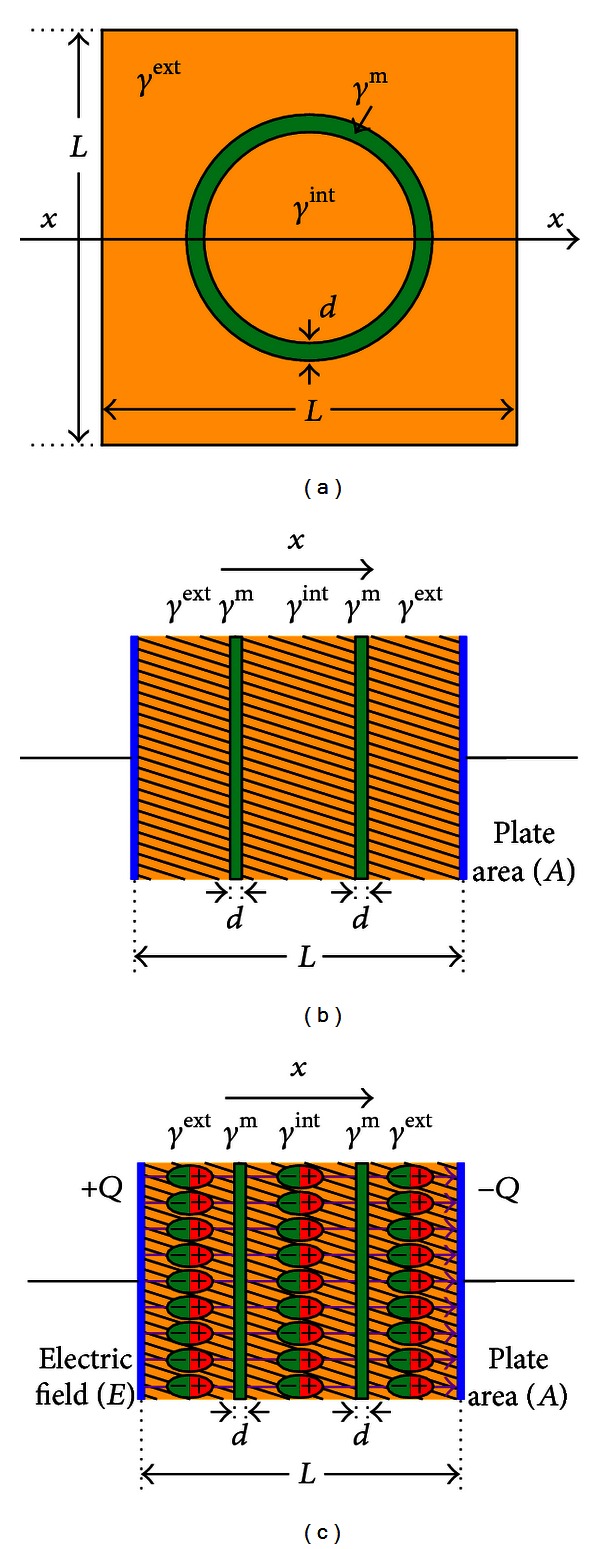
Dielectric phenomena inside a dilute single suspension of a thin membrane from Fricke's model (a) Fricke's model of a dilute single suspension of a thin membrane of thickness *d*, (b) 1D representation of the a dilute single suspension of a thin membrane from Fricke's model of a dilute single suspension of a thin membrane of thickness *d*, (c) dielectric polarization under an electric field *E* within a dilute single suspension of a thin membrane of thickness *d* in 1D.

 Finally, consider the case of a dilute single suspension of membranes (Figure 8) to investigate the role of the thin insulating membrane influencing the frequency-dependent behavior of the effective admittivity. In the model comprising a suspended membrane, there exists a thin membrane *ℳ*_*d*_ of a thickness *d*, as shown in Figure 6, within our target voxel. Assume that the admittivity distribution *γ* changes abruptly across the membrane

(39) $$\begin{array}{c} \gamma \left(r,\omega \right):=\sigma \left(r\right)+i\omega \epsilon \left(r\right)=\{\begin{array}{cc} \sigma^{\mathrm{int}}+i\omega \epsilon^{\mathrm{int}}\;\;\;\; & \text{in}\;\;{ℳ}_{d}\\ \sigma^{\text{ext}}+i\omega \epsilon^{\text{ext}}\;\;\;\; & \text{in}\;\;\Omega \setminus {\overset{-}{ℳ}}_{d}, \end{array}\\ d\approx 0,\;\;\frac{\sigma^{\mathrm{int}}}{\sigma^{\text{ext}}}\approx 0. \end{array}$$

In the case of a dilute suspension of a single membrane, any potential *u* satisfying ∇·(*γ*∇*u*) = 0 can be expressed as

(40) $$\begin{array}{c} u\left(r\right)\approx H\left(r\right)+d\left(\frac{\sigma^{\text{ext}}+i\omega \epsilon^{\text{ext}}}{\sigma^{\mathrm{int}}+i\omega \epsilon^{\mathrm{int}}}\right){𝒟}_{ℳ}\phi \left(r\right), \end{array}$$

where *ℳ* is the surface of the membrane and

(41) $$\begin{array}{c} {𝒟}_{ℳ}\phi \left(r\right)=\int_{ℳ}\frac{\langle r-r^{\prime },n\left(r^{\prime }\right)\rangle }{4\pi {\left|r-r^{\prime }\right|}^{3}}\phi \left(r^{\prime }\right)ds. \end{array}$$

The density *ϕ* is determined by membrane structure and the refraction index:

(42) $$\begin{array}{c} \phi =\frac{\partial u^{\text{ext}}}{\partial n}\;\mathrm{on}\;\;ℳ. \end{array}$$

Here, we recall *𝒟*_*ℳ*_ *ϕ*|_*ℳ*^±^_ = ((±1/2)*I* + *𝒦*)*ϕ* on the surface *ℳ*.

In 1955, Fricke [39] studied the equivalent admittivity for the case of a spherical membrane suspension using its pointwise admittivity distribution (Figure 7):

(43) $$\begin{array}{c} \gamma \left(r,\omega \right)=\{\begin{array}{cc} \gamma^{\mathrm{int}} & \text{if}\;\;\left|r\right|<R-d\\ \gamma^{m} & \mathrm{on}\;\;{ℳ}_{d}=\left\{r:R-d<\left|r\right|<R\right\}\\ \gamma^{\text{ext}} & \text{if}\;\;\left|r\right|>R. \end{array} \end{array}$$

Fricke's expression for the equivalent admittivity for the internal domain including the membrane and *γ* is

(44) $$\begin{array}{c} \gamma \left(r,\omega \right)\approx \{\begin{array}{cc} \begin{array}{c} \gamma_{⋄}^{\mathrm{int}}=\sigma_{⋄}^{\mathrm{int}}+i\omega \epsilon_{⋄}^{\mathrm{int}}\\ =\frac{\gamma^{\mathrm{int}}-\left(2d/R\right)\left(\gamma^{\mathrm{int}}-\gamma^{m}\right)}{\left(1+d/R\right)\left(\left(\gamma^{\mathrm{int}}-\gamma^{m}\right)/\gamma^{m}\right)} \end{array} & \text{if}\;\;\left|r\right|<R\\ \gamma^{\text{ext}} & \text{if}\;\;\left|r\right|>R. \end{array} \end{array}$$

Then the effective admittivity *γ* ^ef^ can be computed by substituting this equivalent admittivity in Maxwell-Wagner-Fricke formula [40]. Under the assumption that three quantities *σ* ^*m*^/*σ* ^ext^,  *σ* ^*m*^/*σ* ^int⁡^, and *d*/*R* are very small, Pauly and Schwan [14] obtained

(45) $$\begin{array}{c} \sigma^{\text{ef}}\left(\omega \right)=\underset{\sigma^{\text{ef}}\left(0\right)}{\underset{︸}{\sigma^{\text{ext}}\left(1-\frac{3c}{2}\right)}}+\underset{\Delta \sigma^{\text{ef}}}{\underset{︸}{\frac{9c\epsilon^{m}}{4\epsilon_{0}}\frac{R}{d\tau }}}\frac{\omega \tau^{2}}{1+\omega^{2}\tau^{2}},\\ \epsilon^{\text{ef}}\left(\omega \right)=\epsilon^{\text{ef}}\left(\infty \right)+\underset{\Delta \epsilon^{\text{ef}}}{\underset{︸}{\frac{9c\epsilon^{m}}{4\epsilon_{0}}\frac{R}{d}}}\frac{1}{1+\omega^{2}\tau^{2}}, \end{array}$$

where

(46) $$\begin{array}{c} \tau =\frac{\epsilon^{m}R}{d}\left(\frac{1}{2\sigma^{\text{ext}}}+\frac{1}{\sigma_{⋄}^{\mathrm{int}}}\right). \end{array}$$

Biological tissues are comprised of cells and an extracellular matrix of macromolecules and extracellular fluid. Cells have different shapes and sizes in the order of *μ*m. The cell has a membrane whose thickness is in the order of several nm. Enclosed within the cell membrane is the cytoplasm containing the nucleus, organelles, and intracellular fluid. Although they are very thin and resistive, cell membranes play an important role in determining admittivity values of tissues. They usually account for large susceptivity values, even though there is little quantitative analysis on how membrane properties affect them.

Membrane suspensions in homogeneous media can be viewed as an equivalent biological tissue admittivity. Analysis of the simple membrane structures should enable us to interpret admittivity images from phantom experiments and numerical simulations.

## 3. Discussion and Conclusions

Tomographic imaging of the admittivity distributions inside biological subjects such as the human body has been an active research goal in electrical impedance tomography (EIT). Can EIT technique distinguish between cucumber and carrot or lung and liver? Measuring the frequency-dependent behavior of effective admittivity increases distinguishability and has a potential of expanding clinical applications. The effective conductivity of biological tissue is associated with the forward problem of an elliptic PDE, the Laplace equation, with a complex material parameters and thin insulating membranes, and there is little understanding about this kind of forward problem. Since traditional electrical impedance tomography can only provide change in effective admittivity on a coarse scale due to its low spatial resolution, we will have a better understanding of EIT images if we can link microscopic cell structures to macroscopic (or effective) tissue admittivity images obtained from multifrequency EIT systems.

We defined the effective admittivity of a cubic voxel from its pointwise admittivity and the Maxwell equations. Because of the reciprocity property, the effective admittivity must be symmetric. Direct computation of effective admittivity using simple models produced the Maxwell-Wagner interface effect and Debye relaxation. The complex permittivity, dielectric constant, and loss factors were derived and modeled as the functions of frequencies, and the dielectric polarization, dispersions, and the relaxation time were studied in a mathematical framework. The single and double layer potentials were used to present a mathematical expression for the Maxwell-Wagner-Fricke expression for the subject containing various geometries of cells or membranes while previous work of Maxwell and Wagner only handles ellipses in a cube.
